# Maternal Mortality among Tribal Women at a Tertiary Level of Care in Bastar, Chhattisgarh

**DOI:** 10.5539/gjhs.v4n2p132

**Published:** 2012-03-01

**Authors:** Prabha Chauhan, V. K. S. Chauhan, Praveen Shrivastava

**Affiliations:** Department of Obstetrics and Gynecology, Government Medical College Jagdalpur (Bastar), Chhattisgarh - PIN- 494001, India Tel: 91-942-559-6754 E-mail: chaupra@rediffmail.com; Department of Community Medicine, Government Medical College Jagdalpur (Bastar), Chhattisgarh -PIN- 494001, India Tel: 91-942-559-0031 E-mail: vinodkschauhan@rediffmail.com; Department of Community Medicine, Government Medical College Jagdalpur (Bastar), Chhattisgarh- PIN- 494001, India Tel: 91-942-553-6441

**Keywords:** Maternal Mortality, Primigravida, Hypertensive Disorders of Pregnancy, Rupture Uterus, Severe Anemia, Tribal women of Bastar, Chhattisgarh

## Abstract

**Objectives:**

The primary objective of this study is to study Maternal Mortality as per Gravidity among Tribal women at a tertiary level of care in Bastar, Chhattisgarh, India.

**Materials and Methods:**

This is a hospital based, retrospective, reproductive-age mortality study (RAMOS) of tribal women of Bastar region, Chhattisgarh, that were admitted and managed in Obstetrics and Gynecology Department Govt. Medical College, Jagdalpur, Bastar, Chhattisgarh, between July 2007 and October 2011. There were total 120 cases.

**Result:**

Results of the present study showed that among 120 deceased tribal women highest maternal mortality 65 cases (54.166%) was noted in Primigravida (Nullipara G1P0), second highest maternal mortality 44 cases (38.333%) was noted in 2^nd^ to 4^th^ Gravida (Multipara), 10 cases (8.333%) were in 6^th^ and 7^th^ Grand Multigravida (Grand Multipara), and 01 case (0.833%) was in 8^th^ Great Grand Multigravida. Direct causes of maternal mortality were highest 46 cases (38.333%) due to hypertensive disorders of pregnancy. Among direct causes second highest 18 cases (14.999%) maternal mortality were due to Rupture Uterus, third highest 12 cases (09.999%) of Septicemia, 06 cases (04.999%) of obstructed labor, 06 (04.999%) of Hemorrhage, 02 cases (01.666%) of unsafe Abortion, 02 cases (01.666%) of Pulmonary Embolism and 01 case (0.833%) due to Aspiration. Indirect causes of maternal mortality maximum 15 cases (12.5%) of Malaria and 10 cases (08.333%) were due to Anemia and 02 cases (01.666%) were of Sickle cell Anemia. The result of the present study showed that in tertiary level of care of Bastar in the year 2007 – 2008, 2008 - 2009, 2009 – 2010 and 2010 - 2011 the total maternal deaths were 34 (n=34); 35 (n=35); 27 (n=27) and 26 (n=26) respectively. The Maternal Mortality Ratio was 1611.876; 1615.881; 1168.325 and 1000.769 Per 1, 00,000 live births in the year 2007- 2008; 2008 - 2009; 2009 – 2010 and 2010 - 2011 respectively. In the year 2007 - 2008, maternal mortality percentage among tribal women was 80.314%; in the year 2008-2009 was 85.714% and was 100% in the year 2009 – 2010 and 2010 – 2011.

**Conclusion:**

Discusses and/or relates this study’s results to the need for improvement in the maternal health of tribal women of Bastar. It has been discussed well in the conclusion section.

## 1. Introduction

*Definition:* Maternal death is the death of a woman while pregnant or within 42 days of termination of pregnancy, irrespective of the duration and site of the pregnancy, from any cause related to or aggravated by the pregnancy or its management but not from accidental or incidental causes. To facilitate the identification of maternal deaths in circumstances in which cause of death attribution is inadequate, a new category has been introduced: Pregnancy-related death is defined as the death of a woman while pregnant or within 42 days of termination of pregnancy, irrespective of the cause of death.

Generally there is a distinction between a direct maternal death that is the result of a complication of the pregnancy, delivery, or their management, and an indirect maternal death that is a pregnancy-related death in a patient with a preexisting or newly developed health problem. Other fatalities during but unrelated to a pregnancy are termed *accidental*, *incidental*, or non obstetrical maternal deaths. Maternal mortality is a sentinel event to assess the quality of a health care system.

Maternal Mortality Ratio is the ratio of the number of maternal deaths per 100,000 live births. The MMR is used as a measure of the quality of a health care system. Sierra Leone has the highest maternal death rate at 2,000, and Afghanistan has the second highest maternal death rate at 1900 maternal deaths per 100,000 live births, reported by the UN based on 2000 figures. According to the Central Asia Health Review, Afghanistan’s maternal mortality rate was 1,600 in 2007. Lowest rates included Ireland at 0 per 100,000 and Austria at 4 per 100,000. In the United States, the maternal death rate was 11 maternal deaths per 100,000 live births in 2005. This rose to 13.3 per 100,000 in 2006.

Maternal Mortality Ratio (MMR); In INDIA TOTAL: Among women aged between 15-49 years dying due to maternal causes per 1, 00,000 live births in 2004-2006 was 254 and in 2007-2009 was 212. In the state of Assam in 2004-2006 and 2007-2009, 480 and 390, in Bihar/Jharkhand 312 and 261, in Madhya Pradesh/ Chhattisgarh 335 and 269, in Orissa 303 and 258, in Rajasthan 388 and 318, in Utter Pradesh/Uttarakhand 440 and 359, in EAG (Empowered Action Group States) and ASSAM SUBTOTAL: 375 and 308, in Andhra Pradesh 154 and 134, in Karnataka 213 and 178, in Kerala 95 and 81, in Tamil Nadu 111 and 97, in SOUTH SUBTOTAL: 149 and 127, in Gujarat 160 and 148, in Haryana 186 and 153, in Maharashtra 130 and 104, in Punjab 192 and 172, in West Bengal 141 and 145, in Other 206 and 160, in OTHER SUBTOTAL: 174 and 149; in 2004-06 and 2007-09, respectively.

## 2. Demographic, Socio-economic and Health Profile of Chhattisgarh State

Total Population (Census 2001) (in millions) 20.83, Crude Birth Rate (SRS 2008) 26.1, Crude Death Rate (SRS 2008) 8.1, Total Fertility Rate (SRS 2008) 3.0, Infant Mortality Rate (SRS 2008) 57, Maternal Mortality Ratio (SRS 2004 - 2006) 335, Sex Ratio (Census 2001) 989, Schedule Caste population (in millions) 2.42, Schedule Tribe population (in millions) 6.62, Female Literacy Rate (Census 2001) (%) 51.9.

## 3. Demographics of District Bastar

“Maternal Mortality among Tribal women at a tertiary level of care in Bastar region” is basically study of “Chhattisgarh state, India” has been purposely add so that viewers can localize the “Bastar”. The data presented here are not representative of India as a whole. “Baster” in Chhattisgarh has been chosen because majority of the population in Bastar is of tribal’s. “According to census of India 2001 basic data sheet of district Bastar; total Schedule Tribe population was 866,488 and 66.31% to total population”.

According to the 2011 census Bastar district has a population of 1,411,644, roughly equal to the nation of Swaziland or the US state of Hawaii. This gives it a ranking of 348th in India (out of a total of 640). The district has a population density of 140 inhabitants per square kilometer (360/sqmi). Its population growth rate over the decade 2001-2011 was 17.83 %. Bastar has a sex ratio of 1024 females for every 1000 males, and a literacy rate of 54.94. According to census of India 2001 basic data sheet of district Bastar; total *Schedule Tribe population* was 866,488 and 66.31% to total population.

Bastar, the land of tribes and natural resources, is the largest tribal district of the newly formed Indian state of Chhattisgarh. About 70% of the total population of Bastar comprises of tribal, which is 26.76% of the total tribal population of Chhattisgarh. The major tribes of the Bastar region are the Gond, Abhuj Maria, Bhatra, Halbaa, Dhurvaa, Muria and BisonHorn Maria. The Gonds of Bastar are one of the most famous tribes in India, known for their unique Ghotul system of marriages. Gonds are also the largest tribal group of central India in terms of population.

The tribes of Bastar region are known for their unique and distinctive tribal culture and heritage in all over the world. Each tribal group in Bastar has their own distinct culture and enjoys their own unique traditional living styles. Each tribe has developed its own dialects and differs from each other in their costume, eating habits, customs, traditions and even worship different form of god and goddess. A large number of Bastar tribal are still living in deep forests and avoid mixing with outsiders in order to protect their own unique culture. The tribes of Bastar are also known for their colorful festivals and arts and crafts. The Bastar Dussehra is the most famous festival of the region. The tribal of Bastar were also amongst the earliest to work with metal and have expertise in making beautiful figurines of tribal gods, votive animals, oil lamps, carts and animals. Bastar is also blessed with exceptional natural beauty and promises to be a favourite destination for researchers, anthropologists, wildlife enthusiasts and nature lovers.

The predominant tribal population in the 4 tehsils were Murias constituting (42.3% HH (House Hold)) the highest concentration (70.5 %) of which live in Dantewara Tehsil (Now District). This was followed by Gonds (41.4% HH), their highest concentration (73.9 %) found in Narainpur Tehsil (Now District), and other tribal groups like Halbas and Bhatras together constituting 16.3 percent of the households.

The household (HH) is “the basic residential unit in which economic production, consumption, inheritance, child rearing, and shelter are organized and carried out”; [the household] “may or may not be synonymous with family”. The household is the basic unit of analysis in many social, microeconomic and government models. The term refers to all individuals who live in the same dwelling.

Tribal of Bastar, Chhattisgarh, India are known for their unique and distinctive tribal heritage and culture all over the world. Each tribal group of this region has its own distinct culture and enjoys a unique traditional living style. The dialect of each of these tribes differs from the others as do their eating habits, costumes, traditions and customs. Even each of these Bastar Tribes worship different forms of gods and goddesses.

The status of tribal women in patrilineal societies has been observed to be somewhat better that of women in a patrilineal society e.g., their legal status is much higher than that of their counter parts in patrilineal societies and they have a significant role in the tribal economy. However, after a comparative analysis of the various indicators (political organization, religion, ritual practices etc.) among the different tribes of India, it has been observed that the status of tribal women is comparatively lower than that of tribal men. Moreover, the status of tribal women has gone from bad to worse as a result of the impact of social change which has affected the social structure of tribal society.

Maternal mortality was reported to be high among various tribal groups but no exact data could be collected. The chief causes of maternal mortality were found to be unhygienic and primitive practices for parturition. For example, the delivery was conducted by the mother herself in a half squatting position holding a rope tied down from the roof of the hut. This helped her in applying pressure to deliver the child. In complicated labour, obviously it might lead to maternal as well as child mortality. Similar crude birth practices were found to exist in other tribal groups. From the inception of pregnancy to its termination no specific nutritious diet is consumed by women. On the other hand, some pregnant tribal women reduced their food intake because of simple fear of recurrent vomiting and also to ensure that the baby may remain small and the delivery may be easier. The consumption of iron, calcium and vitamins during pregnancy is poor. The habit of taking alcohol during pregnancy has been found to be usual in tribal women and almost all of them are observed to continue their regular activities including strenuous physical work during advanced pregnancy. Numbers of deliveries are conducted at home attended by elderly ladies of the household. No specific precautions are observed at the time of conducting deliveries which resulted in an increased susceptibility to various infections. Services of paramedical staff are secured only in difficult labour cases.

## 4. Material and Method

This is a hospital based, retrospective, reproductive-age mortality study (RAMOS) of tribal women of Bastar region, Chhattisgarh, that were admitted and managed in Obstetrics and gynecology Department Govt. Medical College, Jagdalpur, Bastar, Chhattisgarh, between July 2007 and October 2011. There were total 120 Tribal patient (n=120) and 12 (n=12) with a non-tribal background, admitted and managed in indoor wards between July 2007 and October 2011, and the relevant data was collected from the records of the Department of Obstritics & Gynecology and Medical Records Department (MRD), Government Medical Collage and the associated Maharani Hospital, Jagdalpur (Bastar), Chhattisgarh, All 120 Tribal patient (n=120) have been included in this study and 12 (n=12) with non-tribal background have been excluded.

## 5. Objectives

The primary objective of this study is to study Maternal Mortality among Tribal women as per Gravidity at tertiary level of care in Bastar, Chhattisgarh. This is a hospital based retrospective, reproductive-age mortality study (RAMOS) of tribal women of Bastar region, Chhattisgarh. Total 120 Tribal women that were admitted and managed and died (deceased) in Obstetrics and Gynecology Department Govt. Medical College, Jagdalpur, Bastar, Chhattisgarh, between July 2007 and October 2011.

## 6.

### 6.1 Study Population

The sample consisted of total 120 Tribal women patients that were admitted, managed and died (deceased) in Obstetrics and Gynecology Department Govt. Medical College, Jagdalpur, Bastar, Chhattisgarh, between July 2007 and October 2011who attended tertiary care hospital for medical care between July 2007 and October 2011. Those with a non-tribal background, (n=12) were admitted and managed and died (deceased) in Obstetrics and Gynecology Department Govt. Medical College, Jagdalpur, Bastar, Chhattisgarh, between July 2007 and October 2011, were excluded from the study. Finally 120 (n=120) tribal patients were included in the study.

### 6.2 Sampling

This is a hospital based, retrospective, reproductive-age mortality study (RAMOS) of maternal mortality among tribal women of Bastar region. 120 (n=120) tribal patients patients admitted managed and died in the Department of Obstritics & Gynecology, Government Medical Collage and the associated Maharani Hospital, Jagdalpur (Bastar), Chhattisgarh, between July 2007 and October 2011.

### 6.3

“Maternal Mortality among Tribal women at a tertiary level of care in Bastar” is basically study of Bastar region. “Chhattisgarh state India” has been purposely add so that viewers can localize the “Bastar”. The data presented here are not representative of India as a whole. “Baster” in Chhattisgarh has been chosen because majority of the population in Bastar is of tribals. “According to census of India 2001 basic data sheet of district Bastar; total Schedule Tribe population was 866,488 and 66.31% to total population”.

### 6.4

This is a study of Maternal Mortality among Tribal women of Bastar region that were admitted and managed and died (deceased) in tertiary level of care i.e. in the Department of Obstetrics and Gynecology, Government Medical College Jagdalpur (Bastar), Chhattisgarh. Hence Tribal women of Bastar region that were admitted and managed and died in Obstetrics and Gynecology Department Govt. Medical College, Jagdalpur, Bastar, Chattisgarh, between July 2007 and October 2011 total 120 (n=120) cases were included in this study and 12 (n=12) with non-tribal background were excluded.

### 6.5 Data Collection

There were total 120 Tribal patient (n=120) and 12 (n=12). with a non-tribal background, admitted and managed and died in indoor wards between July 2007 and October 2011, and the relevant data was collected from the records of the Department of Obstritics & Gynecology and Medical Records Department (MRD), Government Medical Collage and the associated Maharani Hospital, Jagdalpur (Bastar), Chhattisgarh, All 120 Tribal patient (n=120) have been included in this study and 12 (n=12) with a non-tribal background have been excluded.

### 6.6 Data Analysis

Results were analyzed by using percentage, ratio and *Chi squire test (χ^2 -^ test) for comparative test*.

## 7. Results

Results of the present study showed that among 120 deceased tribal women highest maternal mortality 65 cases (54.166%) was noted in Primigravida (Nullipara) between age group 18 to 35 years, second highest maternal mortality 44 cases (38.333%) was noted in 2^nd^ to 4^th^ Gravida (Multipara) between age group 22 to 42 years, 10 cases (8.333%) were in 6^th^ and 7^th^ Grand Multigravida (Grand Multipara)) between age group 27 to 35 years, and 01 case (0.833%) was in 8^th^ Great Grand Multigravida, of 25 years. Total Maternal Mortality among Primigravida (Nullipara) to Greatgrand Multigravida (Great Grand Multipara) was noted between 18 to 42 age group.

**Table 1.** 

Gravidity	Age Group	Total Cases	Total Percentage
Primigravida	18 - 35	65	54.16%

2^nd^ Gravida			

3^rd^ Gravida	22 - 42	44	36.66%

4^th^ Gravida			

Grand Multigr- Avida (6^th^ & 7^th^)	22- 40	10	8.33%
Great Garand Multigravida (8^th^)	25	1	0.83%
Total		120	100%

Maternal Mortality in among tribal women as per Gravidity and Age at tertiary level of care

Hours of stay in the tertiary level of care: Out of 120 cases 34 cases (28.33%) died within five hours of admission, 31 cases (25.83%) died within fifteen hours, thus cumulative total 65 cases (54.16%) died within fifteen hours of admission, 17 cases (14.16%) died within twenty-five hours of admission, thus cumulative total 82 cases (68.33%) died within twenty-five hours of admission, 16 cases (13.33%) died within fifty hours, thus cumulative total 98 cases (81.66%) died within fifty hours of admission, remaining 22 cases (18.33%) died within three-hundred-twelve hours, Thus all 120 cases died within three-hundred-twelve hours of admission. Table 5 shows details of hours of hospital stay. TERTIARY HEALTH CARE: A specialized, highly technical level of health care that includes diagnosis and treatment of disease and disability in sophisticated large research and teaching hospitals.

**Table 2:** 

S.No.	Duration Range Inhours	Case	Total Cases	Percentage	Cases Cumulative Total	Percentage
1	0.05 – 01 Hrs	9				

2	1.05 – 02 Hrs	6	34	28.333%		

3	2.15 – 05 Hrs	19			65	54.166%

4	5.20-10 Hrs	16				

5	10.15-15Hrs	15	31	25.833%		

6	15.10 -25.10Hrs	17	17	14.166%	82	68.333%

7	26.15 -35.40 Hrs				

8	36 - 50 Hrs	8	16	13.333%	98	81.666%

8	36 – 50 Hrs	8				

9	50 – 64 Hrs	7				

10	66.45-81.2 Hrs	9				

11	83.2 -105.4 Hrs	3	22	18.333%	120	100%

12	135 – 312 Hrs	3				

	TOTAL	120	120	100%	120	

Hours of stay in the tertiary level of care: Duration of management of pregnancy at tertiary level of care

In India managed care is provided free of cost to BPL (Below Poverty Line) poor people. All tribal women included in this study received managed care.

Among 120 cases 99 (82.49%) cases were Ante Natal Cases (ANC) and rest 21 (17.49%) cases were Home Delivery; Post Natal Cases (PNC). On admission among 99 Ante Natal Cases 54.55% (54) cases were in Full Term (36 weeks) gestation, 10.10% (10) cases were in 34 weeks gestation, 13.13% (13) cases were in 32 week gestation, 1.01% (01) case in 31 week gestation, 07% 07 cases in 30 weeks gestation, 11.11% (11) cases in 28 weeks gestation, 3.03% (03) in 24 weeks gestation,. All these 120 cases were unbooked cases and their Ante Natal Check up was not carried out earlier before admission.

**Figure 1:**
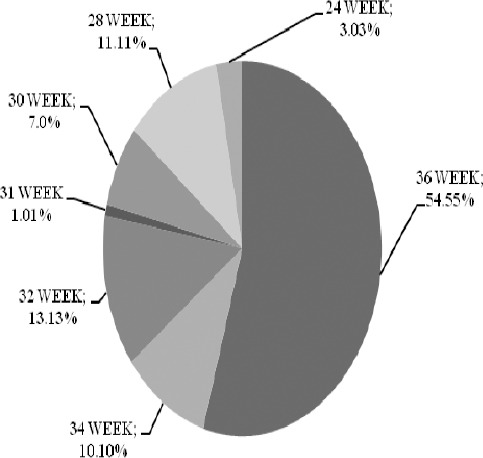
Gestation in which women began perinatal care at tertiary level of care

In Tertiary level of care of Bastar in the year 2007 – 2008, 2008 - 2009, 2009 – 2010 and 2010 - 2011 the total maternal deaths were 34 (n=34); 35 (n=35); 27 (n=27) and 26 (n=26) respectively. The Maternal Mortality Ratio was 1611.876; 1615.881; 1168.325 and 1000.769 Per 1, 00,000 live births in the year 2007- 2008; 2008 - 2009; 2009 – 2010 and 2010 - 2011 respectively. In the year 2007 - 2008, maternal mortality percentage among tribal women was 80.314%; in the year 2008-2009 was 85.714% and was 100% in the year 2009 – 2010 and 2010 – 2011. After analysis of the table: 3 using *Chi squire test (χ^2^ - test)* it is found that the taken variables between Tribal and Nontribal women are highly significant.

**Table 3:** 

Year	2007 - 2008		2008 – 2009		2009 - 2010		2010 - 2011	
MMR(Maternal Mortality Ratio) Per 100000 Live Births	1611.876		1615.881		1168.325		1000.769	

	Among Tribal women	Among Other Cast women	Among Tribal women	Among Other Cast women	Among Tribal women	Among Other Cast women	Among Tribal women	Among Other Cast women

Maternal Mortality Percentage	80.31%	19.68%	85.71%	14.286%	100%	0%	100%	0%

(χ^2^ =38.99) Highly Significant Maternal Mortality Ratio (Mmr)/ Mortality Percentage In Tribal Womennon Tribal Women At Tertiary Level Of Care In Bastar, Chattisgarh

Direct causes of maternal mortality were highest 46 cases (38.333%) due to hypertensive disorders of pregnancy. Out of these 46 cases maximum 30 cases (25%) were due to Eclampsia, second highest 11 cases (09.166%) were due to Pregnancy Induced Hypertension and 05 cases (04.166%) were due to Preeclampsia. Among direct causes second highest 18 cases (14.999%) maternal mortality were due to Rupture Uterus, among these 18 cases of Rupture Uterus, no case was with history of previous caesarean section, third highest 12 cases (09.999%) of Septicemia, 06 cases (04.999%) of obstructed labor, 06 (04.999%) of Hemorrhage, 02 cases (01.666%) of unsafe Abortion, 02 cases (01.666%) of Pulmonary Embolism and 01 case (0.833%) due to Aspiration. Indirect causes of maternal mortality maximum 15 cases (12.5%) of Malaria and 10 cases (08.333%) were due to Anemia and 02 (01.666%) cases were of Sickle Cell Anemia.

**Figure 2:**
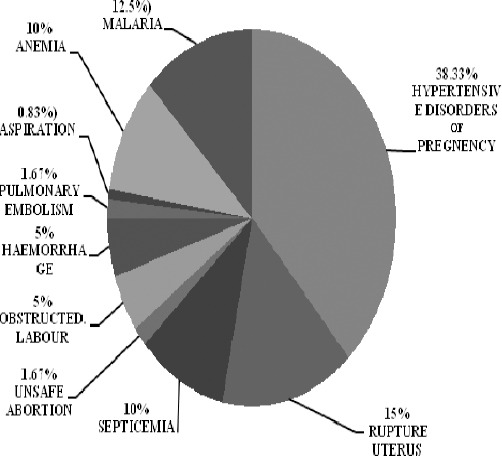
Direct and Indirect causes of Maternal Mortality in tertiary level of care

From Apr. 2007 to Mar. 2008; total (ANC) Ante Natal Cases registered in Bastar region were 61809. Out of these 41950 (67.87%) were Schedule Tribe (ST) women and rest 19859 (32.13%) cases were women of nontribal background. Among these 41950 Schedule Tribe (ST) women; 6418 (15.3%) delivered in tertiary level of care (Obstetrics and Gynecology Department, Government Medical College, Jagdalpur, Bastar); 25673 (61.2%) delivered in other health facilities (Institutional Deliveries) by skilled Birth Attendants; 7971 (19%) Delivered in Home by Trained Birth Attendants (TBA); 1888 (4.5%) Other home deliveries conducted by untrained birth attendants.

From Apr. 2008 to Mar. 2009 total ANC cases registered in Bastar region were 66806. Out of these 47633 (71.3%) were Schedule Tribe (ST) women and rest 19173 (28.7%) cases were women of nontribal background. Among these 47633 Schedule Tribe (ST) women; 8002 (16.8%) delivered in tertiary level of care (Obstetrics and Gynecology Department, Government Medical College, Jagdalpur Bastar); 29580 (62.1%) delivered in other health facilities (Institutional Deliveries) by skilled Birth Attendants.; 8860 (18.6%) Delivery conducted at Home by Trained Birth Attendants (TBA); 1191 (2.5%) Other home deliveries conducted by untrained birth attendants.

From Apr. 2009 to Mar. 2010 total ANC cases registered in Bastar region were 74602. Out of these 51028 (68.4%) were Schedule Tribe (ST) women and rest 23574 (31.6%) cases were women of nontribal background. Among these 51028 Schedule Tribe (ST) women; 8113 (15.9%) delivered in tertiary level of care (Obstetrics and Gynecology Department, Government Medical College, Jagdalpur Bastar); 33832 (66.3%) delivered in other health facilities (Institutional Deliveries) by skilled Birth Attendants.; 8063 (15.8%) Delivery conducted at Home by Trained Birth Attendants (TBA); 1020 (2%) Other Home deliveries conducted by untrained birth attendants.

From Apr. 2010 to Mar. 2011 total ANC cases registered in Bastar region were 78807. Out of these 55323 (70.2%) were Schedule Tribe (ST) women and rest 23484 (29.8%) cases were women of nontribal background. Among these 55323 Schedule Tribe (ST) women; 9239 (16.7%) delivered in tertiary level of care (Obstetrics and Gynecology Department, Government Medical College, Jagdalpur Bastar); 37288 (67.4%) delivered at other health facilities (Institutional Deliveries) by skilled Birth Attendants; 8132 (14.7%) Delivery conducted at home by Trained Birth Attendants (TBA); 664 (1.2%) Other home deliveries conducted by untrained birth attendants.

From Apr. 2011 to Jan. 2012 total ANC cases registered in Bastar region were 65203. Out of these 46555 (71.4%) were Schedule Tribe (ST) women and rest 18654 (28.6%) cases were women of nontribal background. Among these 46555 Schedule Tribe (ST) women; 7356 (15.8%) delivered in tertiary level of care (Obstetrics and Gynecology Department, Government Medical College, Jagdalpur Bastar); 32030 (68.8%) delivered in other health facilities (Institutional Deliveries) by skilled Birth Attendants.; 6937 (14.9%) Delivery conducted at home by Trained Birth Attendants (TBA); 232 (0.5%) Other home deliveries conducted by untrained birth attendants.

## 8. Discussion

According to UNICEF’s “State of the World’s Children-2009” report, 47% of India’s women aged 20–24 were married before the legal age of 18, with 56% marrying before age 18 in rural areas. The report also showed that 40% of the world’s child marriages occur in India.

After analysis of the Table 3 (Chi squire test (χ^2^ – test)) it is found that the taken variables between Tribal and Nontribal women are highly significant

Child bearing imposes additional health needs and problems on women - physically, psychologically and socially. Maternal mortality was reported to be high among various tribal groups. The chief causes of maternal mortality were found to be unhygienic and primitive practices for parturition. For example, it was observed that the delivery was conducted by the mother herself in a half squatting position holding a rope tied down from the roof of the hut. This helped her in applying pressure to deliver the child. In complicated labor, obviously it might lead to maternal as well as child mortality. From the inception of pregnancy to its termination, no specific nutritious diet is consumed by women. On the other hand, some pregnant tribal women reduced their food intake because of simple fear of recurrent vomiting and also to ensure that the baby may remain small and the delivery may be easier. The consumption of iron, calcium and vitamins during pregnancy is poor. The habit of taking alcohol during pregnancy has been found to be usual in tribal women and almost all of them are observed to continue their regular activities including hard labor during advanced pregnancy. Numbers of deliveries are conducted at home attended by elderly ladies of the household. No specific precautions are observed at the time of conducting deliveries which resulted in an increased susceptibility to various infections. Services of paramedical staff are secured only in difficult labor cases.

Pregnancy-induced hypertension and eclampsia are far more common in first pregnancies than in subsequent ones. The relation to age is complex. Most cases of hypertension and eclampsia appear in young women because most primipara is young. Among primipara, there is a U-shaped relation between incidence and age, with far higher rates in older women than in women between the ages of 20 and 30 years. Teenagers appear to have a minimally higher risk of hypertension and ecalmpsia than do women in their 20s. There is also an increase in risk with increasing age among multipara.

Although rates of eclampsia and preeclampsia are higher in blacks than in whites living in industrialized countries, the incidence of these conditions appears to vary little by social class within ethnic groups. In contrast, social class is clearly related to perinatal outcomes of preeclamptic pregnancies. The absence of a relation between incidence and social class is inconsistent with a nutritional or dietary cause of preeclampsia and eclampsia.

The search for nutritional factors associated with preeclampsia is hindered by the lack of data. The review by Green covers this material through the 1980s. Many nutritional factors have been suggested as possible causes of preeclampsia and eclampsia, and there have been many attempts at prevention using dietary or nutritional interventions. No dietary interventions have had much, if any, effect. Many nutrients, including thiamine and sodium chloride, were either not proven to be consistently related or were not studied thoroughly enough to be nominated for programmatic testing and application. Calcium, zinc, and magnesium are 3 micronutrients that seemed very promising, although their roles in preventing preeclampsia and eclampsia are now uncertain.

A majority were cases of unscarred uterus presenting with rupture; the most common cause of rupture in all cases was inappropriate injections of Oxytocin, followed by obstructed labour. All were anemic and most of them were in shock. Conclusion: The leading cause of ruptured uterus was found to be mismanagement by traditional birth attendants. We can reduce maternal mortality due to rupture uterus by giving proper training to traditional birth attendants and by mass education through electronic media.

Anaemia in pregnancy is present in very high percentage of pregnant women in India. Exact data is not available about the prevalence of nutritional anaemia. However according to WHO, the prevalence of Anemia in pregnancy in south East Asia is around 56%. In India incidence of anaemia pregnancy has been noted as high as 40-80%. Risk factors: Sociodemographic factors (age, level of formal education, marital status, areas and cities of residence) Obstetrical factors (gravidity, parity, history of previous preterm or Small-for gestational-age deliveries, plurality of pregnancy multiple or singleton) Behavioural factors (smoking or tobacco usage, alcohol usage, utilization of prenatal care services) Medical conditions (diabetes, renal or cardio-respiratory diseases, chronic hypertension AIP anemia in pregnancy.

Evidence indicates that increasing hookworm infection intensity is associated with lower hemoglobin levels in pregnant women in poor countries.

## 9. Conclusion

From a Maternal Mortality Rate (MMR) of 437 per 100,000 live births in 1990-91, India is required to reduce MMR to 109 per 100,000 live births by 2015. Between 1990 and 2006, there has been some improvement in the Maternal Mortality Rate (MMR) which has declined to 254 per 100,000 live births as compared to 327 in 1990. However despite this progress, India is expected to fall short of the 2015 target by 26 points. Safe motherhood depends on the delivery by trained personnel, particularly through institutional facilities. However delivery in institutional facilities has risen slowly from 26% in 1992-93 to 47% in 2007-08. Consequently, deliveries by skilled personnel have increased at the same pace, from 33% to 52% in the same period. By 2015 India is expected to be able to ensure only 62% of births occur in institutional facilities with trained personnel. Thus universal coverage remains to be achieved.

The problems of Tribal women in India are due to deep rooted community traditions, custom, culture, beliefs and taboos. They are imposed on them by the family, society and community at different levels. It can be said that the socio-economic status of the tribal in Bastar is far below the national standard. The low socio-economic condition is associated with poverty, lack of awareness about personal hygiene, health care & nutrition and livelihood skills to increase productivity using local resources. It has been shared. All these put their development and multidimensional progress and above all, health at risk.

It has been argued by the authors that the improving the “Standard of living” will bring improvement in the health status of Tribal women and Tribal population as a whole. The interaction between social factors and health issues is complex and sometimes unpredictable. For example, in Western Europe during the nineteenth century, increase in income and wealth, resulting from the Industrial Revolution, was accompanied by decrease in both birth and death rates. Many authors have in fact argued that increased income was the main cause of these changes. The situation in the developing world has varied and differs from the so-called “demographic transition” in Europe. In many parts of Asia, and to a certain extent in Latin America, death rates, particularly among infants, have declined steadily in the past decade and birth rates have declined rather dramatically. Yet the increase in income has been very modest. In Africa, on the whole, death rates, particularly of infants, remain high, birth rates are not declining, the benefits of increased income are not yet apparent, and concern over population growth is just emerging. The relationship between wealth, birth, and death rates observed in the development of West European Countries is thus obviously not universal.

The involvement of tribal community in health care delivery system is essential to improve the health status of tribal women of Bastar. A “holistic” approach is needed to organize the health care delivery system in the way it caters the essentials for women of all tribal groups, with emphasis on improvement of the health of tribal women. For example: Planning of health programme according to felt needs of the tribal women groups; IEC programme in their local dialect by the tribal women in connection with nutrition for all category of tribal women with emphasis on pregnant women; Awareness generation for conduction of deliveries by trained staff; discard the old primitive method of parturition by untrained traditional birth attendants, avoid consumption of alcohol, Abstain from use of smokeless tobacco and smoking tobacco, strenuous physical exertion and taking proper rest during pregnancy. Training of health care staff of peripheral health facilities regarding 100% registration of pregnant tribal women and regular Anti Natal Check up, giving Prophylactic/Therapeutic treatment of Anemia during pregnancy; immunization against tetanus, detection of dangerous signs and timely referral to First Referral Units or Tertiary care health facilities; detection of STD’s and identification of Genetic disorders i.e. sickle cell and Glucose-6-Phosphate Dehydrogenase Enzyme Deficiency (G-6-PD) and to provide postnatal health services to all tribal mothers. The above has been shared.

One of 20 case studies in *Millions Saved: Proven Successes in Public Health* is devoted to the reduction of maternal mortality in Sri Lanka. Since 1950, Sri Lanka has reduced maternal deaths “from between 500 and 600 maternal deaths per 100,000 live births in 1950 to 60 per 100,000.” Levine (2007) attributes this decline to four major factors:

1) Broad, free access to a strong health system.
2) The professionalization and broad use of midwives.
3) Gathering of health information and use of this information for policy making.
4) Targeted quality improvements to vulnerable groups.

Sri Lanka accomplished its large reduction in maternal mortality while spending a smaller percentage of GDP on health than most countries at its income level. Maternal mortality decreased more rapidly than female death rates in general. Also, death rates from specific causes of maternal mortality, such as hypertensive disease and sepsis, fell. This suggests that maternal mortality fell due to factors other than general improvements in health. If India and Chhattisgarh state public health services can plan to implement these four factors, India along with Chhattisgarh state will achieve reduction in maternal mortality.
